# Preferred M2 Polarization by ASC-Based Hydrogel Accelerated Angiogenesis and Myogenesis in Volumetric Muscle Loss Rats

**DOI:** 10.1155/2017/2896874

**Published:** 2017-06-14

**Authors:** Hong Huang, Jiejie Liu, Haojie Hao, Deyun Chen, Ling Zhizhong, Meirong Li, Haijing Song, Rong Xiang, Chaoguang Jiang, Xiaobing Fu, Weidong Han

**Affiliations:** ^1^Medical School of Nankai University, Tianjin 300071, China; ^2^Molecular & Immunological Department, Bio-therapeutic Department, Chinese PLA General Hospital, Beijing 100853, China; ^3^Burns Institute, The First Affiliated Hospital of Chinese PLA General Hospital, Beijing 100853, China

## Abstract

Volumetric muscle loss (VML) injury resulted from massive muscle defects and diseases for which there are still no effective therapeutic treatments. This study aimed to investigate the effects of rat adipose-derived mesenchymal stem cells (rASCs) and rASCs-conditioned medium- (CM-) based type I collagen hydrogel on macrophage (MP) transition, myogenesis, and vascularization in the rat VML model. Laser Doppler results demonstrated much higher blood flow in the rASC- and CM-based hydrogel groups. qRT-PCR, hematoxylin and eosin, immunofluorescence, and Sirius Red staining manifested that both rASCs and CM-based hydrogel implantation accelerated muscle repair with upregulated angiogenesis and myogenesis, attenuated inflammation while facilitating M2 transition, and decreased the collagen deposition compared with the hydrogel group. In vitro experiments indicated that factors secreted from polarized M2 MPs could accelerate the migration and tube formation capacities of HUVECs. These results suggested that rASCs exerted immunomodulatory effects on MPs which further enhanced the proangiogenic potential on ECs to promote myogenesis and angiogenesis during muscle repair. These fundamental results support further clinical applications of ASCs for muscle loss injury.

## 1. Introduction

Volumetric muscle loss (VML) is an injury that results from traumatic or surgical actions, which lead to loss of muscle mass and deprivation of function [1]. A large amount of skeletal muscle loss and the destruction of native tissue structures, including the regenerative reservoir, can lead to infection, necrosis, and morbidity [2–4]. Current therapies, including surgical graft with muscle flaps, physical therapy, and rehabilitation, cannot effectively recover the function. However, donor tissue limitations and foreign body reactions challenge muscle flap graft surgeries and prognosis [1, 3, 5]. In this context, the urgent need to decrease the extent of morbidity and disability is required.

Stem cell-based therapy has recently provided a promising way of regenerating skeletal muscle. Adipose-derived stem cells (ASCs) have been widely applied in regeneration medicine [6–10]. Human ASCs (hASCs) took myogenic transition when treated with myogenic differentiation medium or cocultured with myoblasts, which indicated that hASCs possess myogenic potential in vitro [11, 12]. Additionally, local ASC injection to Duchenne muscular dystrophy mice was found to improve muscle regeneration while decrease fibrosis through cellular fusion and downregulated transforming growth factor-*β* (TGF-*β*) [13–15]. However, stem cell injection alone was not sufficient for muscle regeneration and functional restoration in large muscle loss injury. Moreover, stem cell-based tissue engineering (TE) therapy could be an unprecedented option for VML injury treatment [16]. Collagen was the main content of the extracellular matrix (ECM) and has been widely used in tissue engineering and regeneration medicine [17, 18]. In a study on diabetic rabbit ulcers, both the collagen scaffold and the mesenchymal stem cell- (MSC-) seeded collagen benefitted wound closure by accelerating the vascular supply [19, 20]. Kubo and his colleagues reported that type I collagen promoted rabbit articular cartilage healing through providing structure for cell recruitment, cell growth, and proliferation of the host tissue cells [21]. Whether and how to apply ASCs in type I collagen hydrogel to improve angiogenesis and myogenesis in the rat VML injury still needs to be explored.

There is accumulated evidence to show that the immunoregulation and tropical effects were mainly responsible for the stem cell-based TE therapy in muscle repair in animals and preclinical experiments. Since biomaterial worked as a molecular effector, it participated in cellular behavior regulation, such as macrophage (MP) phenotype transition. MPs were essential for muscle repair from the initial debriding of necrosis tissues to the later remolding of neonatal muscle fibers in immunodeficiency mice [22]. Furthermore, it has been demonstrated that in notexin-induced muscle injury mice, proinflammatory M1 acted as activating satellite cells and promoting the proliferation and migration of myogenic cells [23]. While in the late inflammation stage, failure M1 shifted into M2 would hinder the differentiation and delay myogenesis with disorganized ECM deposition in ischemic/reperfusion muscle [24, 25]. In addition, prohealing M2 MPs recently showed a facilitating factor for tissue angiogenesis via secreting vascular endothelial growth factor (VEGF), interleukin (IL) 10, and TGF-*β* in a myocardial infarction model as angiogenesis was critical for nutrient supply and regeneration support during the entire repair process [26, 27]. However, the underlying mechanisms among ASC-based type I collagen hydrogel, MP polarization, angiogenesis, and fibrosis remain to be elucidated in the VML injury model.

Together with these properties, we exogenously implanted rASCs- and its conditioned medium- (CM-) based collagen hydrogel to a rat tibialis anterior (TA) VML model. We observed that both rASCs- and CM-based hydrogel promoted myogenesis, decreased adipose tissue formation, and significantly promoted vascularization. In vivo and in vitro results suggested that MP polarization supported superior blood supply in the defected muscle and EC migration and tube formation. The results provided the basis of rASCs-based hydrogel therapy in the VML injury.

## 2. Materials and Methods

### 2.1. Animals

Male Sprague-Dawley (SD) rats were used in this study and obtained from the Chinese PLA General Hospital (Beijing, China). During the experiments, the rats were housed under standard conditions with a 12 h light/dark cycle. Water and diet were available ad libitum. All procedures were approved by the Institutional Animal Care and Use Committee and the Chinese PLA General Hospital, and all experiments were carried out following the guidelines of the China Council on Animal Care and Use.

### 2.2. Stem Cell Isolation, Culture, and Identification

rASCs were isolated from the rats weighing 60 g~80 g. The inguinal fat tissues were mechanically dissected into pieces, washed three times in phosphate buffered-saline (PBS, Gibco), and then digested by collagenase I (Sigma) and trypsin (Hyclone) in 37°C for 40 min. After filtration through a 100 *μ*m cell strainer and resuspended in DMEM (Hyclone) containing 10% fetal bovine serum (FBS) and 1% antibiotics (Hyclone), the cell cultures were incubated in 37°C. The culture media were changed every 3 days, and rASCs applied for all experimental use were passage 3 or 4.

The multidifferentiation abilities were verified by inducing differentiation assay (Cyagen). rASCs were seeded at 2 × 10^4^/cm^2^ in 6-well cell culture plates in the presence of adipogenic, chondrogenic, or osteogenic differentiation mediums according to the manufacturer's instructions. The induction medium was changed every 3 d for 3 w. After removing the induction medium and fixing by 4% formaldehyde solution, the cells were stained by freshly prepared Oil red O, Alcian blue, and Alizarin red working solutions for specially stained adipocytes, chondrocytes, and osteocytes, respectively. All the results were observed on an inverted-phase contrast microscope (Olympus).

The characteristic surface markers of rASCs were analyzed on the FACScan flow cytometry (BD). The third passage of rASCs was trypsinized, washed once, resuspended in PBS, and incubated with 5 *μ*L monoclonal antibodies at 4°C avoiding light: fluorescein isothiocyanate- (FITC-) conjugated CD45, phycoerythrin- (PE-) conjugated CD31, FITC-conjugated CD34, FITC-conjugated CD44, PerCP-conjugated CD90.1, AF647-conjugated CD29, and Ig isotype antibodies were applied to set the fluorescence background. All the antibodies used for surface marker analysis were purchased from BD.

### 2.3. rASC-Conditioned Medium (rASC-CM) and Mice Macrophage-Conditioned Medium Harvests

The rASC-CM was harvested from the supernatants when cultured in serum-free medium for 24 h, and then the possible cell debris (800 g for 10 min) was collected and removed. The conditioned medium was further concentrated to 10 times by centrifugal filter ultrafiltration (Millipore) and followed by filtration through a 0.22 *μ*m filter. The 10x CM was stored at −80°C before use. Rat VEGF was released from ASC-loaded hydrogel and measured by ELISA kit (Multi Sciences) according to the manufacturers' instructions.

The mice bone marrow-derived cells were aspirated in Hank's solution and centrifuged at 800g for 5 min. Then, the cells were suspended in RPMI-1640 (Gibco) containing 10% FBS, 1% antibiotics, and 100 ng/mL M-CSF (R&D Systems) seeded on 6-well cell plates for culture. The culture media were changed every 3 days. M2 MP activation was stimulated by 20 ng/mL IL-4 (R&D Systems) on 7 d for 36 h. The M2-conditioned medium (M2-CM) were harvested from the supernatants for 36 h, and the possible cell debris (800 g for 10 min) was removed. After filtration, the 1x M2-CM was stored at −80°C before use. Mice MCP-1, IL 10, and IL 8 from M2-CM were examined by ELISA (Multi Sciences).

### 2.4. Type I Collagen Hydrogel Fabrication and Live-Dead Assay

For tracing the distribution, rASCs were labeled with CM-Dil (Invitrogen) in accordance with the instructions of the manufacturer before transplantation. In brief, rASCs were washed twice with PBS, suspended in CM-Dil dilution (10 *μ*mol/L in DMEM), and incubated at 37°C for 5 min and followed by 4°C for 15 min. After incubation, CM-Dil-labeled rASCs were washed twice again and blended with type I collagen (final concentration 2 mg/mL, Solarbio) prior to transplantation. 0.4 mL collagen hydrogel was loaded with 1 × 10^6^ rASCs. The identical volume of rASC-CM was blended with type I collagen as the Hyd-CM group. The type I collagen hydrogel was transplanted as a control group.

The live-dead assay kit (Sigma) was applied for detecting the cell viability according to the instructions of manufacturer. The rASCs at passage three were seeded in type I collagen hydrogel (2 mg/mL, Solarbio) and cultured in 5% CO_2_ at 37°C with 10% FBS and 1% antibiotics. After culture for 10 days, calcein-AM and ethidium homodimer-1 were added to each well, incubated for 15 min, and washed with PBS. Live cells have shown green fluorescence while dead cells have shown red fluorescence. Images were captured through a fluorescence microscope (Olympus), and ten pictures were taken.

### 2.5. Experimental Design

A total of 65 male SD rats with VML injury were randomly assigned to three groups for repairing: collagen hydrogel only (hydrogel), rASC-based hydrogel (Hyd-ASCs), and rASC-conditioned medium-based hydrogel group (Hyd-CM). At 5 d, 1 w, 2 w, 4 w, and 8 w, the rats were sacrificed and the TA muscles were harvested for histological and molecular analyses (*n* = 3 per group). At 2 w after injury, the rats underwent laser Doppler perfusion imaging (LDPI) for perfusion tests to ascertain the therapeutic effects of implantation.

### 2.6. Animal Volumetric Muscle Loss Model

The procedure of creating VML injury in the rat TA muscle was briefly depicted as below. Under aseptic conditions, a lengthwise incision was made to the skin, and a surgical defect of ~10 × 5 × 3 mm (length × width × depth) was created in the middle third of the TA muscle by a scalpel in the right lower leg. All the defected wound beds received Hyd-ASCs, Hyd-CM, or hydrogel grafts. Before replacing by the grafts, the wounds were sutured with simple interrupted Vicryl sutures. The excised defect muscle weight approximated 20% of the estimated total TA muscle. When the hydrogel rASCs and hydrogel CM and collagen hydrogel were shaped under pH 7, they were carefully transplanted to the defected tibia anterior muscle and followed by the stitching and closing of the skin.

### 2.7. Immunofluorescence Staining

**Figure 1:**
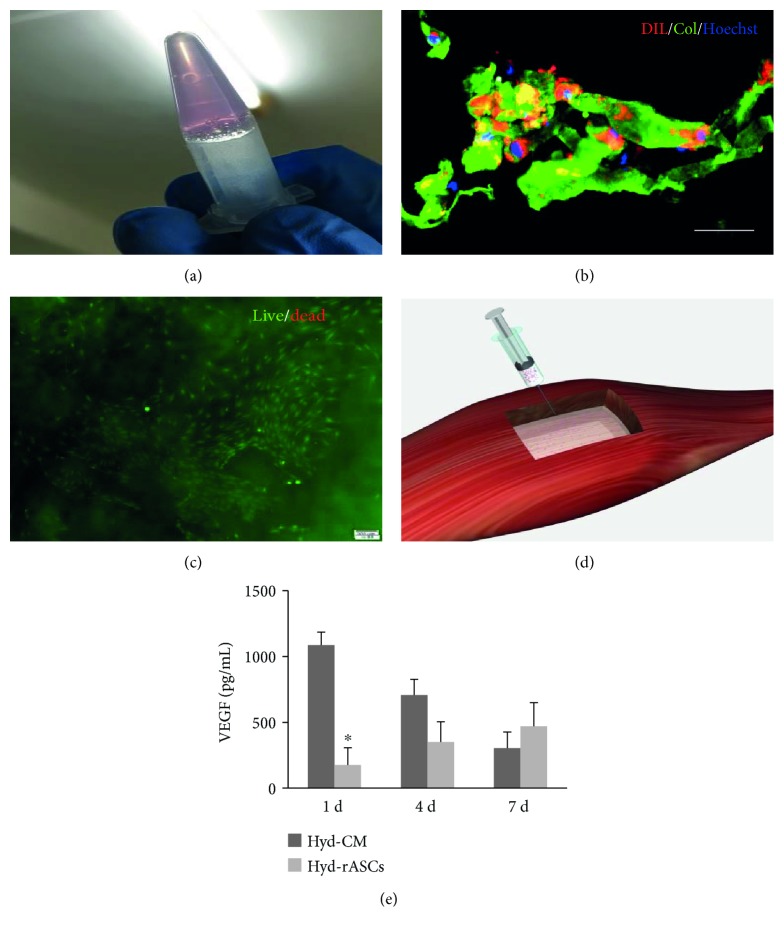
The characterization of injectable hydrogel and rASC viability after seeding. (a) Fabrication of collagen hydrogels. (b) The morphological appearance of CM-Dil-labeled rASCs seeded on collagen hydrogel after 10 days. Scale bar = 20 *μ*m. Green positive represents collagen, red represents CM-Dil-labeled rASCs, and blue represents nuclei. (c) Live/dead cell analysis for ASCs seeded on collagen scaffolds after 10 d seeding. Image displays live cells (green) and dead cells (red). Scale bar = 200 *μ*m. (d) The schematic picture of transplantation. (e) VEGF release in Hyd-CM and Hyd-ASCs on 1 d, 4 d, and 7 d. The experiments were carried out with 4 replicates and data presented as mean ± SD. ^∗^*p* < 0.05 versus Hyd-CM group.

Four muscle sections of each tissue were examined. Two sections were from each side of the TA muscle and the other two sections from the center of the muscle. For morphology observation, Dil-labeled ASC hydrogel was sectioned at 7 *μ*m and stained with collagen I (1 : 400, BOSTER) at 4°C overnight and then incubated with Alexa Flour® 488-conjugated anti-rabbit second antibody, while the nuclei were costained with Hoechst 33342 (Sigma). Then, pictures were visualized under a fluorescent microscope (Olympus), and ten pictures were taken and one represented picture is shown in Figure 1(b).

Muscle tissues were fixed by 4% paraformaldehyde followed by an OCT compound (Sakura Finetek) embedding. 7 *μ*m of the muscle sections was incubated with *α*-SMA (1 : 300, Cell Signaling Technology), and the nuclei were costained with Hoechst 33342. The vascular endothelial cells and CM-Dil-labeled rASCs were imaged via a fluorescent microscope. Two sets of three representative images of each TA muscle were obtained.

To evaluate the effects of Hyd-ASCs and Hyd-CM on macrophage phenotype transition, nonlabeled rASCs and rASC-CM loaded hydrogel were transplanted to VML rats. Then, excised muscle tissues were incubated with the pan-macrophage marker CD68 (1 : 500, Abcam) and the M2 marker Arg-1 (1 : 300, Abcam) to determine the M2 macrophage ratio at the defected area on 5 d and 7 d. The fluorescent images were taken by an Olympus microscope. For quantification, 10 different fields of each muscle sample were randomly selected and Arg I+/CD68+ cell ratio was calculated by Photoshop. The positive staining of each rat was calculated by five slices of four fields each for analysis.

The M2 macrophage induction was confirmed by IF staining of M2 marker Fizz1 (1 : 300, Abcam). The nuclei were costained with Hoechst 33342, and the pictures were taken by a fluorescent microscope.

### 2.8. Laser Doppler Perfusion Imaging (LDPI)

LDPI was carried out to exhibit transplantation-induced changes in tissue perfusion using the LDPI system (Periscan PIM 3 system). Before analyzing, the TA muscle areas of the rats were shaved to expose the injured parts. The rats were sedated and placed on a heating pad in the supine position. Blood perfusion was displayed and ranged from red to dark blue to show high perfusion and low perfusion. LDPI of the muscle was recorded for 3 times at 0 min, 5 min, and 10 min. And blood flow recovery was quantified by the mean pixel value within the ROI, and the value presented as the ratio of the left (defect)/right (normal) LDPI.

### 2.9. Western Blot

The lysates of muscle tissue samples on 1 w post injury were loaded on SDS-PAGE gel and transferred to the PVDF membrane. Then CD68 (1 : 1000, Abcam), iNOS (1 : 1000, Abcam), ArgI (1 : 1000, Abcam), TNF-*α* (1 : 1000, Abcam), *α*-SMA (1 : 1000, Cell Signaling Technology), desmin (1 : 50,000, Abcam), fibromodulin (FMOD, 1 : 1000, Abcam), myogenin (1 : 1000, Abcam), MyoD (1 : 100, Santa Cruz Biotech), collagen I (1 : 1000, BOSTER), HGF (1 : 100, Santa Cruz Biotech), AKT (1 : 1000, Cell Signaling Technology), pAKT (1 : 1000, Cell Signaling Technology), and GAPDH (1 : 20,000, Santa Cruz Biotech) were probed with the membrane as the primary antibodies at 4°C overnight. The membranes were rinsed and incubated with anti-mouse or anti-rabbit second antibodies at room temperature for 50 min. The blots were visualized by exposure to chemiluminescence ECL reagents (Beyotime). The gray values of the target bands were calculated by Image J software. Each result was obtained by at least three independent experiments.

### 2.10. qRT-PCR

To quantify the tissue repair and regeneration-related gene expressions at the mRNA level, the qRT-PCR method was applied. TA muscle tissues were harvested from the rats on the indicated days, and the total RNA was extracted by the TRIzol agent (Invitrogen) and transcribed into cDNA (Invitrogen). Then, qRT-PCR was performed in 30 *μ*L of THUNDERBIRD™ SYBR Green qPCR mixture (TOYOBO) on the ABI PRISM 7500 Real Time PCR System (Applied Biosystems). The primer sequences used in this study are shown in Supplementary Table 1 available online at https://doi.org/10.1155/2017/2896874. The relative expression of target mRNA was normalized to the internal control 18S and analyzed by the ΔΔC_t_ method. All results were repeated at least three times.

### 2.11. Histology Examination

The TA muscle samples were obtained at the indicated times, were fixed by neutral formalin, and embedded with paraffin. All samples were processed and sectioned at 7 *μ*m thick; the sections were performed with hematoxylin and eosin (H&E), Sirius Red, and Masson's trichrome staining. The collagen deposition area and ratio was quantified by Image Pro Plus 6.0 software.

### 2.12. Scratch Assay and Tube Formation Assay

The scratch assay was applied to assess the migration effect of M2 macrophage-secreted factors to the endothelial cells. HUVECs were seeded in 6-well plates until they were fully confluent. The wound healing was scraped by the 200 *μ*L pipette tip on the bottom wells. The medium was then changed into a serum-free culture medium supplemented with 1, 10, 20, 50, and 100 *μ*L M2-CM. By the time of 0 h and 24 h, representative five pictures of each scratch were taken to quantify the migration ability by Image J software (NIH). The vascular tube formation assay was performed by seeding the HUVECs in a 12-well plate precoated with Matrigel™ (BD), adding different concentrations of M2-CM and incubating the plates for 18 h at 37°C. The number of capillary-like structures was quantified and visualized under an inverted phase-contrast microscope (Leica, Germany).

### 2.13. Statistical Analysis

All results were presented as mean ± standard error of the mean (SEM). Differences between groups were examined for statistical significance using Student's *t*-test and analysis of variance (ANOVA). Statistical significance was defined when *p* < 0.05. Statistical analysis was carried out by using SPSS 11.0 for Windows (SPSS Inc.) or the GraphPad Prism 5.0 software (GraphPad Software Inc.).

## 3. Results

### 3.1. rASC Isolation and Identification In Vitro

**Figure 2:**
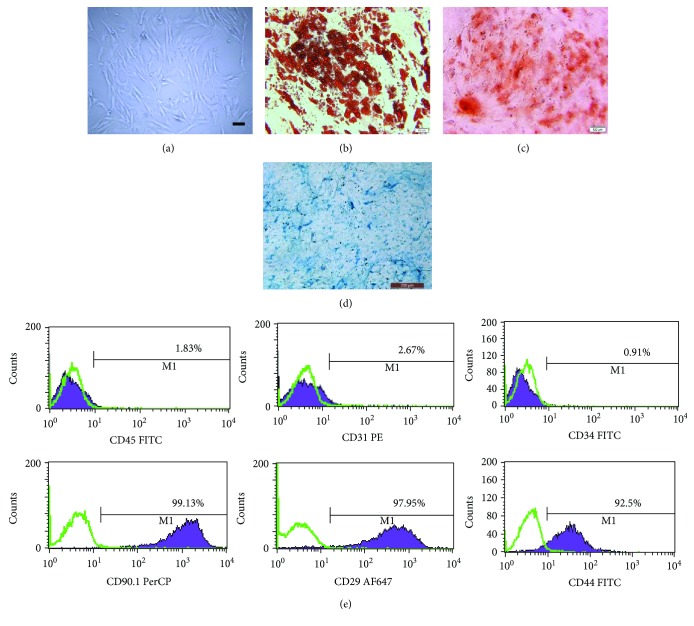
Characterization of rat adipose-derived mesenchymal stem cells (rASCs). (a) Passage 3 of cultured rASCs under light microscope. Representative pictures of (b) adipogenic, (c) osteogenic, and (d) chondrogenic induction for 3 w of rASCs followed by Oil red O, Alizarin red, and Alcian blue staining. Scale bar = 100 *μ*m. (e) Flow cytometry analysis of the cell surface markers CD45, CD31, CD34, CD90.1, CD29, and CD44 expression at passage 3.

To identify the immunophenotype of isolated rASCs, the rASCs of passage 3 underwent multipotential differentiation toward the adipogenic, chondrogenic, and osteogenic lineages after 3 w inductions (Figures 2(a)–2(d)). Meanwhile, we performed FCM to analyze the expression of typical markers (Figure 2(e)). The surface markers' profile showed 1.83%, 2.67%, and 0.91% of CD45, CD31, and CD34, respectively, and 99.13%, 97.95%, and 92.5% of CD90.1, CD 29, and CD44, respectively. This expression pattern was in accordance with the studies previously reported [19, 28].

### 3.2. Fabrication of Collagen Hydrogel and Transplantation

1 × 10^6^ rASCs labeled with CM-Dil or were not blended with 0.4 mL type I collagen (final concentration 2 mg/mL) prior to transplantation. The identical volume of rASC-CM was blended with collagen as the Hyd-CM group. When the hydrogel rASC and hydrogel CM were shaped under pH 7, they were carefully transplanted to the defected tibia anterior muscle (Figures 1(a) and 1(d)). The morphology of labeled rASCs was observed at 10 d (Figure 1(b)). Survival of rASCs after seeded in type I collagen hydrogel was stained by live-dead assay at 10 d (Figure 1(c)). And VEGF secreted by ASCs and released by ASCs was measured by ELISA on 1 d, 4 d, and 7 d (Figure 1(e)). An obvious decrease of VEGF release was observed in the Hyd-CM group while Hyd-ASCs constantly produce VEGF. It is suggested that rASCs seeded in hydrogel still maintain survival and secretory capabilities.

### 3.3. rASC-Based Hydrogel Accelerating Muscle Regeneration by Promoting Angiogenesis and Myogenesis While Attenuating Collagen and Adipose Deposition

**Figure 3:**
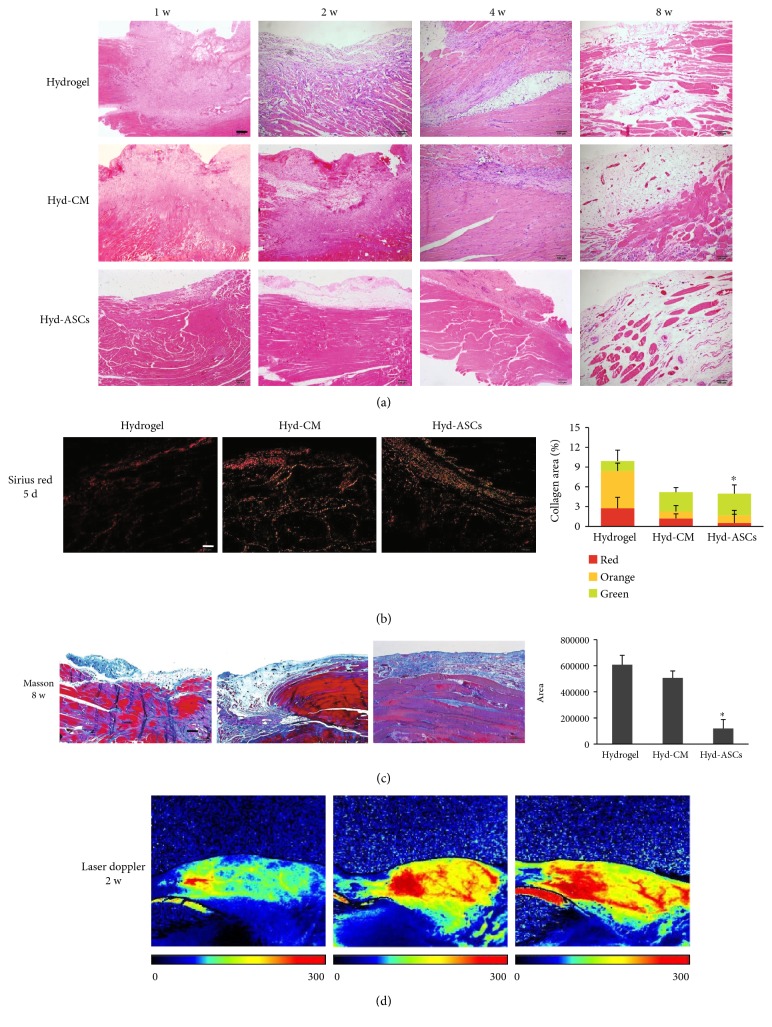
rASC-based hydrogel accelerating muscle regeneration. (a) H&E staining of TA muscle during repair and regeneration at 1 w, 2 w, 4 w, and 8 w post injury. Scale bar = 100 *μ*m. (b) Sirius Red staining at 5 d post injury and collagen analyzed. Scale bar = 100 *μ*m. (c) Masson trichrome staining of TA muscle at 8 w post injury and collagen analyzed. Scale bar = 200 *μ*m. (d) Laser Doppler perfusion images at 2 w post injury. Images above were representatives of at least three independent experiments. Data were mean ± SEM of three independent experiments. ^∗^*p* < 0.05 versus hydrogel group.

A total of 65 male SD rats with VML injury were randomly assigned to three groups for repairing: collagen hydrogel only (hydrogel), rASC-based hydrogel (Hyd-ASCs), and rASC-conditioned medium-based hydrogel group (Hyd-CM). To evaluate the muscle repair outcomes of rASC-based hydrogel treatment, the rats were sacrificed at indicated time. Then, muscle sample from defected area was harvested and H&E, Masson trichrome, and Sirius Red staining were performed at 5 d, 1 w, 2 w, and 4 w. H&E staining displayed that inflammation still existed at 1 w. Continued myogenesis was observed at 4 w and 8 w, while the hydrogel group appeared to have accumulative adipogenesis, which would impede muscle repair (Figure 3(a)). Sirius Red stain at 5 d showed that significant decline in total collagen deposition was displayed (9.91 ± 0.23%, 5.09 ± 0.16%, and 4.694 ± 0.25%) (*p* < 0.05) (Figure 3(b)). Masson trichrome staining at 8 w showed that there was a much stronger blue-stained collagen deposition in the hydrogel group than in the Hyd-rASCs and Hyd-CM groups (Figure 3(c)). The collagen area of the three groups was 607,776 ± 576, 505,975 ± 673, and 11,926 ± 904, respectively. LDPI tests on 2 w showed significantly higher blood flow restoration in the Hyd-rASCs and Hyd-CM treatment groups, while there was no obvious improvement in the hydrogel group 2 w after injury (Figure 3(d), Table 1).

### 3.4. rASC-Based Hydrogel Ameliorated Inflammation and Vasculogenesis during Muscle Regeneration

**Figure 4:**
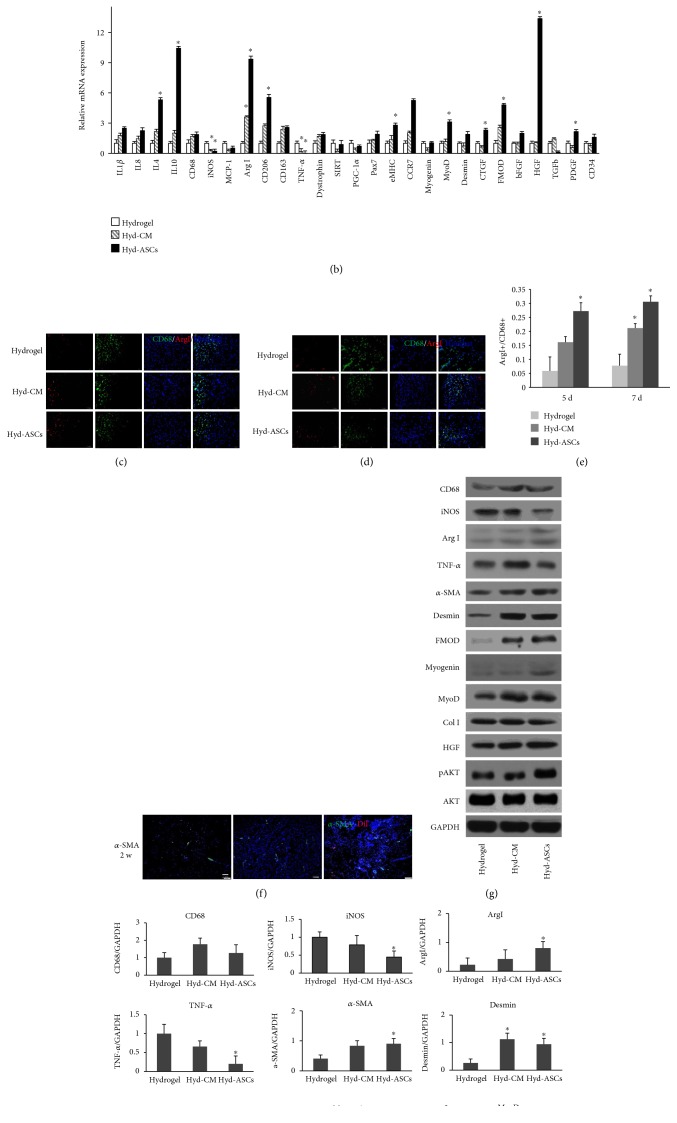
rASC-based hydrogel regulating inflammation, myogenesis, and vasculogenesis during muscle regeneration. (a) and (b) displayed the inflammation, angiogenic, and myogenic mRNA expression at 5 d and 2 w during muscle repair and regeneration. mRNA expressions were normalized to 18S for rat muscles. (c), (d) IF staining of Arg I (red) and CD68 (green) at 5 d and 7 d in wound areas. (e) Arg I+/CD68+ ratio manifested more prohealing M2 profile in the Hyd-ASC and Hyd-CM groups than in the hydrogel group. Scale bar = 20 *μ*m. (f) The IF staining of *α*-SMA and CM-Dil-labeled ASCs at 2 w post injury. Scale bar = 100 *μ*m. Nuclei in blue, ECs in green, and rASCs in red. (g) The pan macrophage marker CD68, M1 marker iNOS, M2 marker Arg I, endothelial cell marker *α*-SMA, myogenic markers Myogenin and MyoD, other prohealing-related protein like desmin and fibromodulin (FMOD), HGF, collagen I, inflammation protein TNF-*α*, signaling pathway protein AKT, and pAKT expressions were detected by Western blot. GAPDH was loaded as control. (h) Relative protein expression was analyzed by fold of GAPDH or ratio of phosphorylation to total protein. Data were mean ± SEM of three independent experiments. ^∗^*p* < 0.05 versus hydrogel group.

To reveal the mechanisms of ASCs promoting muscle regeneration, we conducted qRT-PCR to examine the inflammation, angiogenesis, and muscle regeneration-related mRNA expression at 5 d and 2 w (Figures 4(a) and 4(b)). The results showed that proinflammatory interleukins, such as IL1*β* and TNF-*α*, and necrosis factors, such as SIRT and PGC-1*α*, dramatically decreased while anti-inflammatory interleukins, such as IL4 and IL10, were much highly elevated. MPs in the injured sites were vitally important in muscle repair and regeneration. CD68 and MCP-1 expressions in the Hyd-ASCs and Hyd-CM groups were higher than those in the hydrogel group, with elevated Arg I, CD206, and CD163 and decreased iNOS. This result indicated that a higher ratio of M2 type MP was expressed in the wound sites. In view of myogenesis, the qRT-PCR results showed that eMHC and CCR7 were much higher than the hydrogel group; myofibril differentiation factors, such as MyoD, dystrophin, and CTGF, were all upregulated at 5 d and 2 w, and other repair-related factors, such as fibromodulin (FMOD), hepatocyte growth factor (HGF), and platelet-derived growth factor (PDGF), were elevated at 5 d and 2 w.

In order to verify if the Hyd-ASCs hydrogel transplantation would alter MP phenotype polarization, Arg I and CD68 underwent immunofluorescence (IF) and were quantified. Costaining Arg I and CD68 manifested that large quantity of macrophages was recruited in the wound sites while higher ratio of M2 macrophages showed in the Hyd-ASCs and Hyd-CM groups compared with the hydrogel group both at 5 d and 7 d (Figures 4(c), 4(d), and 4(e)). The IF staining of vascular marker *α*-SMA at 2 w after injury showed significantly higher angiogenesis in the Hyd-ASCs and Hyd-CM groups than in the hydrogel-treated group (Figure 4(f)). The WB results also affirmed that M2 MP and *α*-SMA expressions were elevated in the Hyd-ASCs and Hyd-CM groups than in the hydrogel group. CD68, iNOS, and TNF-*α* expressions showed that M1 MPs and inflammation were decreased; desmin, HGF, Col I, and FMOD expressions also increased in the Hyd-ASCs and Hyd-CM groups. Myogenesis-related protein Myogenin and MyoD were upregulated in Hyd-ASCs and Hyd-CM groups; meanwhile, the pAKT/AKT ratio elevated in the process of muscle repair and regeneration (Figures 4(g) and 4(h)). These results above indicated that rASCs loaded in collagen hydrogel improved muscle repair and remolding via regulating a series of cytokines and growth factor expression to enhance the macrophage phenotype shift and support angiogenesis.

### 3.5. Polarized M2 Accelerated EC Migration and Tubular Formation

**Figure 5:**
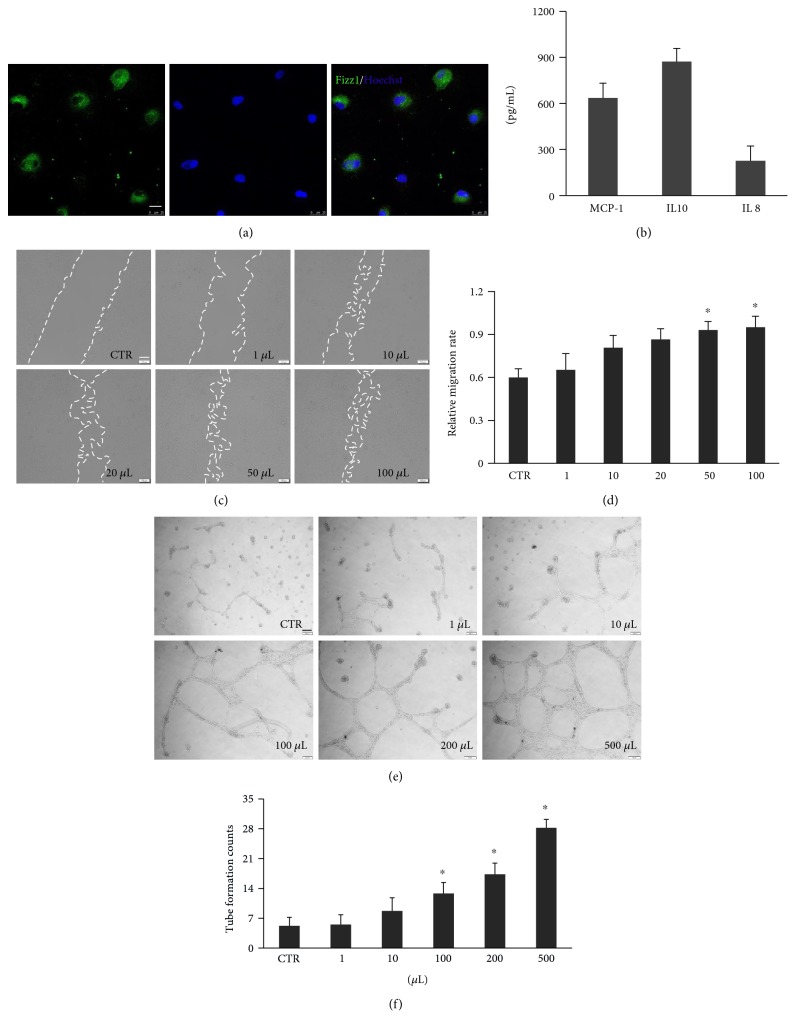
M2-CM enhanced EC vasculogenesis in vitro. (a) M2 macrophage induction identification by IF staining for M2 marker Fizz 1. Scale bar = 20 *μ*m. (b) MCP-1, IL 10, and IL 8 released by M2 macrophage. (c) M2-CM promoted EC migration at 24 h through scratch assay. Scale bar = 100 *μ*m. (d) Analysis of relative migration rate of ECs after M2-CM stimulation for 24 h. (e) Typical images of the tube-like structures in the presence of M2-CM cultured on Matrigel at 18 h. Scale bar = 200 *μ*m. (f) Analysis of tube formation counts of different additions of M2-CM. Data were mean ± SEM of three independent experiments. ^∗^*p* < 0.05 versus CTR group.

M2 macrophage was induced by IL4 stimulation for 36 h, and the M2 MP transition was confirmed by IF staining of Fizz1 (Figure 5(a)). M2 MPs secreted soluble factors in the CM such as MCP-1, IL 10, and IL 8 were evaluated by ELISA (Figure 5(b)). Also, to determine the proangiogenesis ability of M2-CM on the ECs, 1 *μ*L, 10 *μ*L, 20 *μ*L, 50 *μ*L, and 100 *μ*L M2-CM were added to the HUVECs after scratching. After 24 h, the M2-CM significantly accelerated the EC migration (Figures 5(c) and 5(d)). In the tube-like structure formation assay, we found that 100 *μ*L, 200 *μ*L, and 500 *μ*L M2-CM significantly facilitated HUVECs' tubular formation capacities after 18 h stimulation (Figures 5(e) and 5(f)). To conclude, M2 macrophages secreted cytokines to promote EC migration and tube formation.

## 4. Discussion

VML injury is associated with devastating cosmetic and functional deficits. In the present study, we showed that rASC- or rASC-CM-based hydrogel filled the defected TA muscle and displayed effective myogenesis, improved blood perfusion, and decreased excessive collagen deposition compared with the hydrogel-alone group. Improved angiogenesis was mainly attributed to the enhanced release of anti-inflammation cytokines to skew MPs towards a prohealing M2 phenotype as well as angiogenic factors, which led to enhanced EC recruitment and growth. Both immunomodulation and angiogenesis partly accounted for the secretion effects by the engrafted rASC-based hydrogel.

Major contributions of transplanted rASC-based hydrogel to the VML injury rats have been demonstrated by promoting neovascularization and immunoregulation. When rASCs were injected to the cardiotoxin-induced skeletal muscle injury mice, muscle was regenerated with neovascularization and inflammation manipulation [14]. In the present study, the increase in the content of anti-inflammatory factors, such as IL4 and IL10, and M2 MP markers CD163, CD206, and Arg I showed that after rASC- and CM-hydrogel implantation, early protection from muscle defects induced inflammation. With regard to vascularization, the paracrine secretion of angiogenic factors by ASCs also had been reported to be important for enhancing tissue repair [19, 28]. In our study, the mRNA expressions of PDGF and HGF were all increased since day 5. These factors were reported to be potent proangiogenic factors when injected alone, or they enhanced expression in transplanted cells to infarcted myocardium [29–31]. FMOD plays a vital role in reducing scar formation, disorganized collagen deposition via TGF-*β* signaling pathway in wound healing [32]. Both the mRNA and protein expressions of FMOD were upregulated in the present study, indicating aiding muscle repair and reducing scar formation. Furthermore, *α*-SMA protein expressions at 7 d and 2 w, as well as blood flow observation by LDPI, indicated that rASCs significantly accelerated angiogenesis in the wound skeletal muscle. As the rASC-CM-based hydrogel transplantation group also showed better performance than the control group, it indicated that proangiogenic effects were partially via a paracrine mechanism [33]. It was worth to emphasize the continuous release of active growth factors to ameliorate the inflammatory microenvironment and strong angiogenesis by rASC-based hydrogels.

Type I collagen hydrogel is participated in tissue repair and has been documented to be benefitted for the reconstruction of extracellular matrix and connective tissue [34]. Excessive collagen deposition was documented to hinder muscle function recovery. By applying H&E, Masson trichrome, and Sirius Red staining, we observed that fibroblast cells and collagen deposition significantly decreased in rASC- and rASC-CM-based hydrogel groups. Additionally, collagen fibers were more finely arranged in the rASC-treated groups compared with the hydrogel group. All these findings were confirmed by qRT-PCR, lower expressed TGF-*β* at 2 w in rASC- and rASC-CM-based hydrogel, which proved that rASCs owned antifibrosis characteristics in muscle defect injuries. rASCs secreted several cytokines and growth factors with cytoprotective and tissue repair features, including HGF. HGF is a pleiotropic factor that promotes angiogenesis and inhibits fibrosis during wound repair. The mRNA expressed in the rASC- and rASC-CM-based hydrogel was much higher than that in the hydrogel group, which further illustrated that rASCs could reduce the extent of collagen fiber proliferation.

The polarization of MPs matters significantly to the repair of VML injury. MPs participate in both the amplification of inflammation to debride necrotic cells and the regulation of the inflammatory response to avoid excess tissue damages [24]. The proinflammatory M1 shift to the prohealing M2 phenotype promoted the resolution of the tissue inflammation stage and moved toward the remodeling stage [25]. The in vivo experiments had demonstrated that MPs alter their phenotype according to their tissue environment [35, 36]. Nevertheless, different MP phenotypes remolded the environment through secreting series of cytokines to evoke or lock the inflammatory signaling pathways. The M1 MPs acted by satellite cells and promoting the proliferation and migration of myogenic cells; however, they inhibited myoblast differentiation while M2 MPs promoted myocyte differentiation and remolded them into myofibers [23]. Among the cytokines that M2 MPs secreted, anti-inflammatory IL10 was an extremely potent factor that facilitated skeletal muscle regeneration. As our results suggested that the MPs recruited to the sites of defected skeletal muscle played a key role in VML repair. At the mRNA level, the engrafted Hyd-rASC treatment recruited more inflammatory cells (CD68+ cells) at 5 d. They incurred an enhanced M2/M1 ratio at the early time point of 5 d, initiated a tissue repair cascade, and then expeditiously resolved the inflammatory process to further promote muscle repair compared with the hydrogel group. Our findings were upheld that the dynamic immune modulation observed during rASC-based hydrogels mediated muscle repair and is important to properly regenerate skeletal muscle. In the rASC-based hydrogel treated group, muscle differentiation-related mRNAs, such as dystrophin, CCR7, myogenin, MyoD, and CTGF, were upregulated. However, the Hyd-rASC group did not exhibit consistency, which suggested that the secretory effect was not enough for a series of muscle repair events besides angiogenesis. Hence, the prohealing effect imparted by the rASCs in the current study is, at least partially, mediated by the anti-inflammatory factors they secreted, which have been shown to mediate the resolution of inflammation and accelerate myogenesis in vivo. As such, it is worth trying to optimize the rASCs' culture environment to enhance the secretion of myogenic factors.

## 5. Conclusions

We demonstrated that the implantation of ASC-based and CM-based hydrogel accelerated muscle repair and regeneration through partial transdifferentiation into ECs, ameliorating inflammation and increasing the transition of M2 MPs, which are strongly associated with angiogenesis. These fundamental results support further clinical application of ASCs for muscle loss injury.

## Supplementary Material

Supplementary Table 1 The primers used to perform RT-PCR.

## Figures and Tables

**Table 1:** Blood perfusion value in each group (mean ± SD).

Group	*n*	0 min	5 min	10 min
Hydrogel	27	11.47 ± 0.54	10.97 ± 0.79	12.35 ± 0.67
Hyd-CM	27	16.53 ± 0.41	17.12 ± 0.31	16.83 ± 0.46
Hyd-ASCs	27	21.06 ± 0.95^∗^	23.01 ± 0.16^∗^	22.35 ± 0.74^∗^

There is a statistical difference between the hydrogel group and Hyd-ASCs group (^∗^*p* < 0.05).
