# Supplementary material for: Preferred M2 Polarization by ASC-Based Hydrogel Accelerated Angiogenesis and Myogenesis in Volumetric Muscle Loss Rats
Source: Stem Cells Int. 2017 Jun 14;2017:2896874. doi: 10.1155/2017/2896874 (PMC5488492; doi:10.1155/2017/2896874)
Supplement: Supplementary file 1 — Supplementary Table 1 The primers used to perform RT-PCR. [file 2896874.f1.doc]

Supplementary Table 1 The primers used to perform RT-PCR.

| **Gene** | **Forward Sequence** | **Reverse Sequence** | **Length** |
| --- | --- | --- | --- |
| IL 1β | TACCTATGTCTTGCCCGTGGAG | ATCATCCCACGAGTCACAGAGG | 101 |
| IL 4 | TGATGTACCTCCGTGCTTGA | AGGACATGGAAGTGCAGGAC | 197 |
| IL 8 | TGTGGGTCTGTTGTAGGG | CGGTGGTAGAATGGAGTG | 298 |
| IL 10 | CCCAGAAATCAAGGAGCATT | GCTCCACTGCCTTGCTTTTA | 116 |
| TNF-α | TCCGCAGATACCTGGAACTC | CTCAGATCCTCCCCATTCAA | 172 |
| CD68 | TGTACCTGACCCAGGGTGGAA | GAATCCAAAGGTAAGCTGTCCGTAA | 140 |
| CD206 | ACTGCGTGGTGATGAAAGG | TAACCCAGTGGTTGCTCACA | 68 |
| iNOS | CCAACCTGCAGGTCTTCGATG | GTCGATGCACAACTGGGTGAAC | 257 |
| MCP-1 | CGTGCTGTCTCAGCCAGAT | GGATCATCTTGCCAGTGAATG | 71 |
| Arg1 | GTGAAGAACCCACGGTCTGT | GTGAGCATCCACCCAAATG | 180 |
| CD163 | TCATTTCGAAGAAGCCCAAG | CTCCGTGTTTCACTTCCACA | 101 |
| CCR7 | GCTCTCCTGGTCATTTTCCA | AAGCACACCGACTCATACAGG | 107 |
| SIRT | GTTGACCTCCTCATTGTTATTGG | CGCAGTCTCCAAGAAGCTCT | 151 |
| PGC-1α | CGTGTTCCCGATCACCATA | GTGTGCGGTGTCTGTAGTG | 108 |
| FMOD | GAAGATCCCTCCTGTCAACACCAAC | CACCTGCAGCTTGGAGAAGTTCAT | 124 |
| TGF-β | GTCAGACATTCGGGAAGCA | CCAAGGTAACGCCAGGAAT | 138 |
| CTGF | CAAGCAGCTGGGAGAACTG | ACAGGGTGCACCATCTTTG | 127 |
| Pax7 | GCAGTCGGACCACATTCAC | CGCACGACGGTTACTGAAC | 155 |
| eMHC | TGGAGGACCAAATATGAGACG | CACCATCAAGTCCTCCACCT | 180 |
| Myogenin | CTACAGGCCTTGCTCAGCTC | GTTGGGACCAAACTCCAGTG | 153 |
| MyoD | CGTGGCAGTGAGCACTACAG | TGTAGTAGGCGGCGTCGTA | 133 |
| Dystrophin | GCCAAAGTTTGAACCAGGAC | ACAGGCCTTTATGCTCATGC | 183 |
| Desmin | TACACCTGCGAGATTGATGC | TTAGGTGTCGGATCTCCTCCT | 142 |
| HGF | CATTGGTAAAGGAGGCAGCTATAAA | GGATTTCGACAGTAGTTTTCCTGTAGG | 253 |
| bFGF | GCCTTCCCACCCGGCCACTTCAAGG | GCACACACTCCCTTGATGGACACAA | 179 |
| CD34 | ACCTCCAAGCAAAGCAAAGA | GACGGCTGGAGGAGAGTTC | 169 |
| 18S | GGCCCGAAGCGTTTACTT | ACCTCTAGCGGCGCAATAC | 173 |

iNOS, inducible nitric oxide synthase; MCP-1, monocyte chemotactic protein 1; Arg1, Arginase-1; CCR7, C-C chemokine receptor type 7; SIRT, Sirtuin; PGC-1α, peroxisome proliferators activated receptor gamma co-activator 1; FMOD, fibromodulin; TGF-β1, tranforming growth factor-β1; CTGF, connective tissue growth factor; eMHC, embryonic myosin heavy chain; HGF, hepatocyte growth factor; bFGF, basic fibroblast growth factor.
